# Antibiotics Knowledge, Usage, and Prescription Patterns Among Dental Practitioners in Hyderabad, South India

**DOI:** 10.7759/cureus.49554

**Published:** 2023-11-28

**Authors:** Kranti Kiran Reddy Ealla, Neema Kumari, Vikas Sahu, Vishnupriya Veeraraghavan, Palavardhan Peddapalegani, Pratibha Ramani, Srikrishna Sulgodu Ramachandra

**Affiliations:** 1 Oral and Maxillofacial Pathology, Saveetha Institute of Medical And Technical Sciences, Chennai, IND; 2 Oral and Maxillofacial Pathology, Malla Reddy Institute of Dental sciences, Hyderabad, IND; 3 Microbiology, Malla Reddy Institute of Medical Sciences, Hyderabad, IND; 4 Oral and Maxillofacial Pathology, Malla Reddy Institute of Dental Sciences, Hyderabad, IND; 5 Biochemistry, Saveetha Institute of Medical And Technical Sciences, Chennai, IND; 6 Statistics, Sri Venkateswara University, Tirupati, IND; 7 Oral and Maxillofacial Pathology, Saveetha Dental College and Hospitals, Saveetha University, Chennai, IND; 8 Community and Family Medicine, Malla Reddy Medical College for Women, Hyderabad, IND

**Keywords:** dentistry, dental practitioners, kap survey, antimicrobial resistance, antibiotics

## Abstract

Background: Antimicrobial resistance is a pertinent issue in the healthcare sector, accounting for 1.27 million patient deaths worldwide. Dental practitioners account for 3% to 11% of total antibiotic prescriptions. Therefore, this study aimed to specifically assess their knowledge of antibiotic prescriptions, guidelines, and clinical practices.

Method: Before conducting this knowledge, attitude, and practice (KAP) survey, study approval was obtained from the Scientific Review Board of Saveetha Dental College and Hospitals, Chennai, India. A total of 200 participants were randomly selected from the list of Indian Dental Association (Hyderabad chapter), and dental colleges, dental conferences, and peer suggestions. We received a total of 130 responses by the end of the survey.

Results: The survey revealed gaps in practitioners’ KAP. Of those surveyed, 83 (63.85%) of the practitioners kept themselves updated about antibiotic guidelines and news, but many (94, 72.31%) were unaware of the WHO’s access, watch, reserve (AWaRe) and antimicrobial stewardship concepts (103, 79.23%). A total of 111 (85.38%) practitioners considered cross-reactions with other medications, yet only 28 (21.5%) practitioners tested patients for antibiotic sensitivity. While 64 (49.23%) practitioners encountered patients who did not respond to antibiotics, 84 (64.62%) practitioners encountered patients who demanded antibiotics.

Conclusion: This study highlights the lack of awareness about the WHO's AWaRe classification and antimicrobial stewardship among the majority of dental practitioners across Hyderabad. Misuse or overuse of antibiotics was indicated in this survey by both patients and dental practitioners. Prioritizing updates on antibiotic knowledge and guidelines and awareness of their use is important. It is essential to educate patients about the ill effects of self-prescribing antibiotics. Dental practitioners need to consider cross-reactions and antibiotic-sensitivity testing before prescribing antibiotics. Labeling the sensitivity of a particular antibiotic for specific microbes on packaging can help reduce misuse and the use of antibiotics for the wrong indications.

## Introduction

In the healthcare system, antibiotics are a boon to combat infections. However, with this revolutionary advancement, came a worldwide major health concern in the form of antimicrobial resistance (AMR). Yearly, 1.27 million deaths take place worldwide due to AMR [1]. Therefore, a large number of antibiotics are no longer in use due to their lost effectiveness over time.

According to a study, self-prescription of antibiotics has increased by up to 88% in several countries [2]. In India, antibiotics are available over the counter (OTC) at pharmacies without prescriptions. This allows people to self-prescribe the antibiotics. In many instances, antibiotics are over-prescribed. This leads to overuse or misuse of antibiotics, which in turn significantly contribute to AMR, thus making treatment difficult or impossible. For example, AMR has resulted in several strains of *Staphylococcus aureus *being resistant to vancomycin and methicillin [3-5]. Although the development of new medicines helps target resistant bacteria, overuse or unnecessary prescriptions again lead to AMR. Therefore, proper knowledge of antibiotic usage and resistance is mandatory in any medical field.

In the last two decades, the use of systemic antibiotics has increased to a greater extent in dentistry, and it is still common in day-to-day practice. In the healthcare sector, 3% to 11% of total antibiotic prescriptions are from dentistry [6-8] to prevent infections. Dental infections are composed of different types of bacteria, such as gram-positive, gram-negative, and anaerobes [9]. Therefore, dentists use antibiotics as an adjunct to prevent infection at the treatment site. However, not all clinical scenarios involving oral infections require antibiotics. Antibiotics are usually required for medically vulnerable individuals and cases where there is a systemic impact, like malaise, trismus, lymphadenopathy, fever, and ongoing infections. Endodontic conditions like reversible and irreversible pulpitis, pulpal necrosis, acute apical periodontitis, and certain abscesses do not require antibiotic treatment [10]. However, dental practitioners still prescribe antibiotics for these conditions [3,11]. Further, knowledge, attitude, and practice (KAP) surveys conducted among dental practitioners from different places worldwide, such as India (Mumbai [3], West Bengal [12], Jharkhand [13], Davangre [14], and Uttar Pradesh [15]), Spain [16], and the Dominican Republic [11], indicate the lack of an established pattern for antibiotic prescription and misuse by both patients and dental practitioners. Surveys also indicated a lack of awareness among dental practitioners regarding WHO-established guidelines [3]. Therefore, knowledge of proper antibiotic practices by dental practitioners is crucial. In this context, we conducted a KAP survey regarding antibiotics among dental practitioners in Hyderabad, India.

## Materials and methods

The study was conducted to assess the knowledge, attitudes, and practices of dental practitioners regarding the use of antibiotics in their clinical practice. Before carrying forward this KAP survey, approval for the study was obtained from the Scientific Review Board of Saveetha Dental College and Hospitals, Chennai, India (approval no. SRB/SDC/PhD/OPATH-2114/23/117). Following approval, a semi-structured questionnaire was prepared and shared via Google Forms (Alphabet Inc., Mountain View, CA, USA) following a review of existing literature on the topic [3,11,15,17,18]. The survey was conducted between January 2023 and July 2023, involving dental practitioners in Hyderabad (Telangana, India).

The questionnaire was sent to 200 dental practitioners who had either Bachelor of Dental Surgery (BDS) and/or Master of Dental Surgery (MDS) degrees. These practitioners were randomly selected from the list of dentists registered under the Indian Dental Association (Hyderabad chapter), and from dental colleges, dental conferences, and peer suggestions. The number of participants was limited to 200 to ensure the timely completion of the review. They were informed about the purpose of this survey, and after that, they were sent the Google Form. The questionnaire was designed to ensure that the questions were well-informed, comprehensive, and as per the purpose of this study. Some questions were designed after discussion among the authors, and some were referred to from the previous literature by Parekh et al. [3] and Aragoneses et al. [11].

The questionnaire had two parts. The first part focused on general and demographic information about the participating dental practitioners. This section included four questions to collect information regarding gender, age group, qualification, and years of work experience. This information was crucial to establishing a context for the survey and providing a clear understanding of the backgrounds of the participants. The second part of the questionnaire focused on the KAP of antibiotics among dental practitioners. There were 15 questions in this section. The purpose of these questions was to determine the knowledge of participants about antibiotics, their attitude towards antibiotic prescription, and their actual practices concerning antibiotic use in dental treatment. Dental practitioners who were sent the questionnaire were sent two rounds of reminders and followed up via phone call for those who did not respond after the second round of online reminders. A total of 130 practitioners submitted responses. The survey was closed until the end of July 2023 to work on data organization and analysis. All questionnaires were marked as mandatory, so responses to all questions were received. All the responses received from practitioners who responded were included in this study. The responses were arranged in a table. The data were then quantitatively analyzed and plotted in graphs as required.

Statistical analysis

The chi-square test and Fisher’s exact test were conducted for statistical analysis using SPSS Statistics version 22.0 (IBM Corp., Armonk, New York, USA). The p-values were calculated to determine the significance of the results. A p-value of <0.05 was considered statistically significant.

## Results

**Figure 1:**
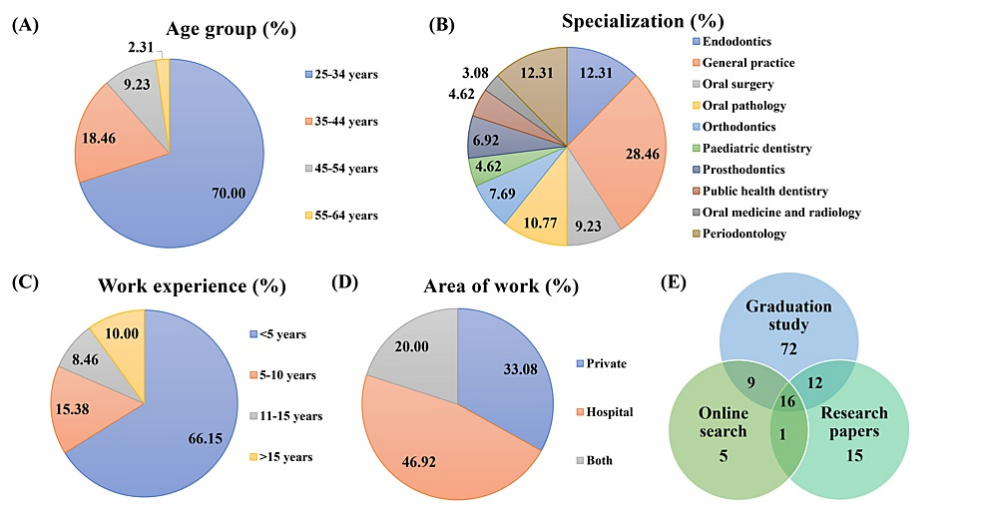
Data about practitioners regarding (A) age group, (B) specialisation, (C) work experience, (D) work area, and (E) source of antibiotic knowledge

Responses were received from 130 practitioners, corresponding to a 65% response rate. Of the 130 practitioners, 84 (64.62%) were female and 46 (35.38%) were male. Forty-nine (37.69%) practitioners had a BDS degree, and 81 (62.31%) practitioners had an MDS degree. Ninety-one (70%) practitioners were in the age group of 25 to 34 years, followed by 24 (18.46%) practitioners in the age group of 35 to 44 years (Figure 1A). From the data presented in Table 1, the majority were general practitioners (37, 28.46%), followed by other practitioners specializing in endodontics (16, 12.31%) and periodontology (16, 12.31%) (Figure 1B). Most of the practitioners (86, 66.15%) had <5 years of work experience (Figure 1C). Responding practitioners were either working in private clinics (43, 33.08%), hospitals (61, 46.92%), or both (26, 20.00%) (Figure 1D). Regarding antibiotic knowledge, practitioners obtained and updated information from their education during graduation, research papers, as well as online searches (Figure 1E).

**Table 1:** Responses of the dental practitioners to questions based on work experience as part of the KAP survey KAP: Knowledge, attitude, and practice

S. no.	Questions		Work experience (N%)				Total
			<5 years	5-10 years	11-15 years	>15 years	
1	Do you believe that the regular prescription of antibiotics without any indication of infection is unnecessary?	Yes	37	11	7	8	63
		No	17	4	2	1	24
		Sometimes	32	5	2	4	43
2	Which is the most prescribed antibiotic by you? [2,8]	Amoxicillin/clavulanic acid	81	19	10	12	122
		Azithromycin	8	1	1	3	13
		Cephalexin	2	0	0	3	5
		Clindamycin	1	0	0	1	2
		Metronidazole	20	3	2	7	32
		Penicillin	6	0	0	0	6
		Other	1 (augmentin 625 mg)	1 (Doxycycline)	0	0	2
3	You prefer to use antibiotics to treat ___.	Primary infection	31	8	3	4	46
		Secondary infection	3	1	1	1	6
		All	52	11	7	8	78
4	What are the conditions in which you generally prescribe antibiotics? [2]	Facial swelling due to dental-related causes	70	17	9	11	107
		Pain relief	20	2	3	1	27
		Implant	39	6	0	5	50
		Periodontal surgeries	47	11	1	8	67
		Endodontic flare-up	26	7	3	3	39
		Reversible pulpitis	9	3	0	1	13
		Irreversible pulpitis	27	4	5	2	38
		Dentoalveolar abscess	61	15	8	10	94
		Facial cellulitis	49	12	7	9	77
		Replantation after avulsion	26	6	3	4	39
		Luxation/subluxation	13	4	1	3	21
5	Do you test a patient for antibiotic sensitivity before prescribing an antibiotic? [2]	Yes	24	1	0	3	28
		No	39	15	7	4	65
		Sometimes	23	4	4	6	37
6	Do you take into account the cross-reaction between the prescribed antibiotics and the other medications prescribed/being taken by the patient?	Yes	73	17	8	13	111
		No	13	3	3	0	19
7	Do patients themselves demand antibiotics sometimes?	Yes	55	16	4	9	84
		No	31	4	7	4	46
8	Have been you visited by patients not responding to antibiotics? [2]	Yes	40	8	6	10	64
		No	46	12	5	3	66
9	Your knowledge of antibiotics is based on ___. [8]	Graduation study	78	17	5	9	109
		Online search	16	8	2	5	31
		Research papers	20	10	8	6	44
10	Do you keep yourself updated about the antibiotics guidelines, news, and updates? [2]	Yes	50	14	8	11	83
		No	36	6	3	2	47
11	Do/Have you attend(ed) programs related to antibiotic usage and resistance? [2]	Yes	25	8	5	7	45
		No	61	12	6	6	85
12	Do you know about the AWaRe classification by WHO to use antibiotics? [2]	Yes	21	6	3	6	36
		No	65	14	8	7	94
13	Do you know about the Antimicrobial Stewardship concept by WHO? [2]	Yes	15	2	4	6	27
		No	71	18	7	7	103
14	Do you think that there is a need to study about antibiotic resistance phenomenon more in-depth?	Yes	75	17	9	13	114
		No	3	1	0	0	4
		Maybe	8	2	2	0	12
15	Should the susceptibility of an antibiotic toward pathogens be labeled on its packet? [2]	Yes	65	14	8	9	96
		No	6	2	0	1	9
		Maybe	15	4	3	3	25

**Figure 2:**
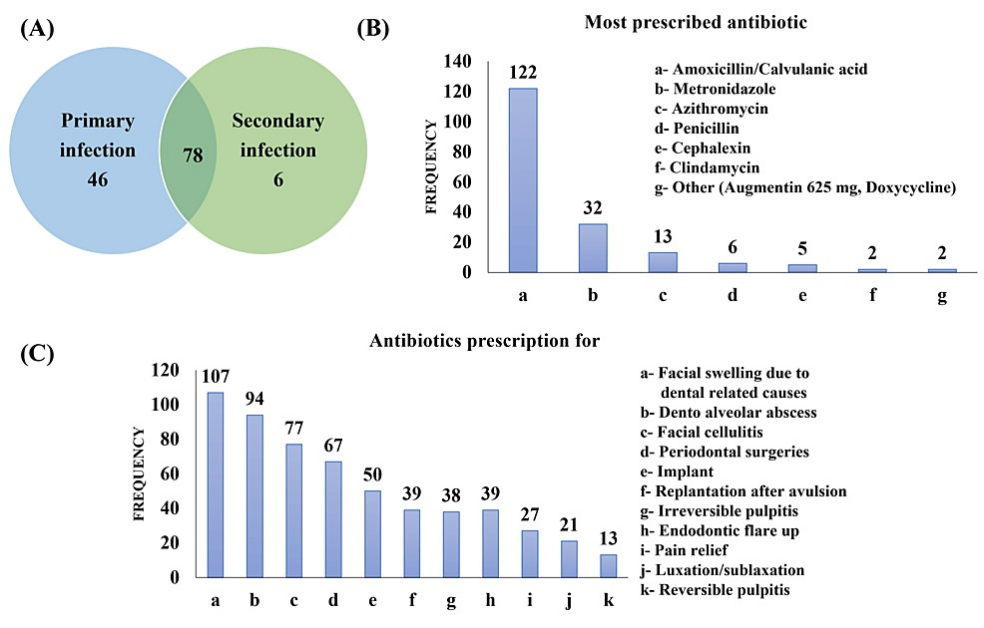
Data regarding (A) antibiotics prescription for the type of infections, (B) antibiotics prescribed by the practitioners, (C) antibiotics prescription for dental conditions

Statistically, there were no significant differences between any groups for any question. Seventy-eight (60%) dental practitioners prescribed antibiotics to treat both primary and secondary types of infections, whereas 46 (35.38%) practitioners prescribed antibiotics only for primary infections (Figure 2A). As for the antibiotics prescribed (Figure 2B), amoxicillin/clavulanic acid (122, 93.38%) was the most prescribed antibiotic, followed by metronidazole (32, 24.62%) and azithromycin (13, 10%). Antibiotics were mostly prescribed (Figure 2C) for facial swelling due to dental causes (107, 82.31%), followed by dentoalveolar abscess (94, 72.31%) and facial cellulitis (77, 59.23%).

Irrespective of work experience, 63 (48.46%) practitioners believed that regular antibiotic prescriptions are unnecessary without any indication of infection, and 43 (33.08%) practitioners believed that it is sometimes unnecessary. Most practitioners (65, 50%) did not test patients for antibiotic sensitivity before prescribing antibiotics, while 28 (21.54%) practitioners reported always testing for antibiotic sensitivity, and 37 (28.46%) practitioners sometimes tested for antibiotic sensitivity. Out of 130 practitioners, 111 (85.38%) also considered cross-reactions of antibiotics with other medications being prescribed or taken by patients. Eighty-four (64.62%) practitioners also received patients who asked for self-prescription of antibiotics, and 64 (49.23%) practitioners also received patients who were not responding to antibiotics.

While 83 (63.85%) practitioners kept themselves updated about antibiotic guidelines and news, only 45 (34.62%) practitioners attended or participated in programs related to antibiotic use and resistance. Most practitioners did not know of the access, watch, reserve (AWaRe) classification (94, 72.31%) and the antimicrobial stewardship concept (103, 79.23%) of the WHO. Overall, 114 (87.69%) practitioners believed that the current knowledge on antibiotic resistance should be further expanded, and 96 (73.85%) practitioners favored the idea of labeling antibiotic packages with the susceptibility to pathogens.

## Discussion

The present study evaluated antibiotic knowledge, attitudes, and practices among dental practitioners in Hyderabad. The survey had a response rate of 65%, similar to the studies by Goud et al. (66.6%) [14] and Segura-Egea et al. (64%) [16]. However, the response rate of this study was much lower than the other studies conducted by Parekh et al. (95%) [3], Mohan et al. (87.3%) [13], and Kumar et al. (87.8%) [9] and higher than the study conducted by Kaul et al. (38.33%) [12]. The relatively small number of participants presents substantial limitations for this study. The small number of participants limits the generalizability of the findings to the larger population of dental practitioners in Hyderabad. Consequently, the conclusions drawn might not accurately represent the diverse practices of the larger dental community. Therefore, to improve the strength and applicability of the study, future research involving larger and more diverse participants is required. A larger sample size with a diverse range of practitioners in terms of experience, specialties, and practice settings could offer a more comprehensive understanding of the subject matter. This expanded scope would likely help draw more robust conclusions, enhancing the credibility and relevance of the findings.

Nevertheless, the majority of responses in this survey were from young dental practitioners aged 25 to 34 years (91, 70%) and with <5 years of work experience (86, 61.5%), similar to the study in West Bengal by Kaul et al., where 71% of participants were below 30 years of age [12]. With 24 (18.46%) dental practitioners believing that regular prescription is not unnecessary and 43 (33.08%) practitioners believing it is unnecessary only sometimes, the study indicates the overprescription of antibiotics by some dental practitioners. Amoxicillin/clavulanic acid is a broad-spectrum antibiotic [19]. Therefore, it is a first-line treatment option for odontogenic infections. Notably, amoxicillin/clavulanic acid, which is the most prescribed antibiotic in the healthcare system, poses a threat of global resistance to it. A large number of participants (122, 93.8%) in this survey prescribed amoxicillin or clavulanic acid. Amoxicillin or clavulanic acid is also found to be the major choice of practitioners from Spain (84.4%) [20] and those in parts of India (Jharkhand (50%) [13], Jammu (37.8%) [21], and Maharashtra (30%) [22]). Conversely, practitioners from other parts of India, such as Mumbai (43.7%) [3], West Bengal (37%) [12], Davangere (33% to 60%) [14], and Uttar Pradesh (71.7%) [15], prescribed amoxicillin more than other antibiotics. The next most prescribed antibiotic among the participants was metronidazole (32, 24.6%), similar to the participants from Mumbai (32.1%) [3] and Uttar Pradesh (33.3%) [15], and much higher than the participants from Spain (0.8% to 13%) [16]. Additionally, 34% of participants from West Bengal prescribed metronidazole along with amoxicillin [12]. In another survey conducted by Kumar et al. with participants from Hyderabad, it was found that the majority of the practitioners prescribed amoxicillin with metronidazole (30%), the next choice of antibiotics was amoxicillin (29.3%), and amoxicillin/clavulanic acid was prescribed by 20.5% of practitioners [9].

Many endodontic conditions, such as reversible pulpitis, irreversible pulpitis, and pain relief, for which antibiotics are prescribed do not require such prescriptions because studies have shown that antibiotics do not provide any useful results in those conditions [12,23-25]. Yet, 27 (20.77%) practitioners prescribe antibiotics for pain relief, 38 (29.23%) for irreversible pulpitis, and 13 (10%) for reversible pulpitis. This result is similar to the results of the survey conducted among the dental practitioners from West Bengal [12] (pain relief (42%), irreversible pulpitis (35%), and reversible pulpitis (13%)), but much higher than the practitioners from Mumbai [3] (pain relief (1.9%), irreversible pulpitis (5.1%), and reversible pulpitis (1.6%)).

Not screening patients for antibiotic sensitivity by 65 (50%) practitioners and sometimes by 37 (28.46%) practitioners is another major flaw in antibiotic prescribing practices. Practitioners (66%) from Mumbai not prescribing antibiotic sensitivity tests indicate that this issue is prevalent outside Hyderabad too. However, examining cross-reactions between antibiotics and other medications prescribed by 111 (85.38%) practitioners indicates the importance given to cross-reactions and awareness of them among practitioners. This awareness is greater among practitioners with more experience, reflecting the role of work experience in instilling cautionary behavior among practitioners. The other major concern comes from patients asking for antibiotics themselves and from patients showing antibiotic resistance. These indicate a lack of awareness about the ill effects of antibiotics among non-medical people who can easily self-prescribe them or get them OTC. Similar cases were seen in Mumbai, where 76.1% of dental practitioners encountered patients who self-prescribed antibiotics [3], compared to the 84 (64.6%) dental practitioners in this survey. The practitioners (51.3%) from Mumbai also encountered patients not responding to antibiotics. Additionally, AMR in patients suggests the development of antibiotic resistance following a prescription or pre-existing resistance. This indicates the need for antibiotic sensitivity testing before prescribing any antibiotic.

Furthermore, the lack of updation among 47 (36.15%) practitioners and 85 (65.38%) practitioners not attending programs on antibiotic guidelines, news, use, and antibiotic resistance is another concern promoting antibiotic misuse. The number of participants in this study who lack awareness about the AWaRe classification (94, 72.31%) and the antimicrobial stewardship concept (103, 79.23%) was lower than the participants from the Mumbai survey (87.2% for AWaRe and 93.3% for antimicrobial stewardship concept) [3]. This lack of awareness among dental practitioners further adds to concerns about antibiotic misuse.

Although 114 (87.69%) practitioners agreed that antibiotic resistance needed to be studied in more depth, 96 (73.85%) practitioners agreed to susceptibility labels on antibiotic packaging. Similarly, 82.4% of practitioners from the Mumbai survey agreed to susceptibility labels on antibiotic packets [3]. Although the number of practitioners agreeing to these was high, many practitioners not or partially agreeing to them is another challenge to bring seriousness to AMR.

Comparative analysis of this survey with others reveals a widespread lack of antibiotic knowledge, poor attitude, and misuse or overuse of antibiotics across various regions in India and beyond. Antibiotic prescriptions by practitioners are often based on multifactorial choices that are not necessarily required. This can further exacerbate the AMR issue. Consequently, proper guidelines and knowledge are required among dental practitioners worldwide to address this growing concern.

## Conclusions

Overall, this survey provided valuable insights about the awareness level of dental practitioners in Hyderabad regarding AMR, though a large number of practitioners still do not consider it a major issue. It is essential to educate patients about the ill effects of self-prescribing antibiotics. Cross-reaction and antibiotic-sensitivity testing need to be considered by dental practitioners before issuing antibiotic prescriptions. Incorporating WHO guidelines into courses and attending seminars describing antibiotic use and WHO guidelines will further improve the knowledge of dental practitioners.
